# Evaluation of Implant Surface Modification with Nanohydroxyapatite Associated with the Use of L-PRF: In Vivo Study in Rats

**DOI:** 10.3390/jfb14070370

**Published:** 2023-07-14

**Authors:** José Augusto Gabarra Júnior, Fernando Nóbrega, Paula Gabriela Oliveira, Edmara Tatiely Bergamo, Uislen Cadore, Milene Zezzi do Valle Gomes, Per Kjellin, Liat Chaushu, Fabio Bezerra, Bruna Ghiraldini, Sergio Scombatti de Souza

**Affiliations:** 1 Department of Oral and Maxillofacial Surgery and Periodontology, School of Dentistry of Ribeirão Preto, University of Sao Paulo, Ribeirão Preto 14040-904, SP, Brazil; 2 Department of Periodontology, School of Dentistry, University Center of State of Para, Belem 66060-575, PA, Brazil; 3 Department of Periodontology and Prosthodontics, Bauru School of Dentistry, University of Sao Paulo, Bauru 17012-901, SP, Brazil; 4 Promimic AB, AstraZeneca BioventureHub, 481 83 Mölndal, Sweden; 5 Department of Periodontology and Implant Dentistry, School of Dentistry, Tel Aviv University, Tel Aviv 6997801, Israel

**Keywords:** dental implant, leukocyte-platelet-rich fibrin, nanohydroxyapatite

## Abstract

Leukocyte–platelet-rich fibrin (L-PRF) contains growth factors that stimulate bone regeneration. This study evaluated the bone repair in a tibia rat model around two implant surfaces in combination or not with L-PRF by assessing microtomographic and histomorphometric parameters. A total of 48 female rats were used in the study, in which 24 received implants with two types of surface treatments (dual acid etched—DAE or nanohydroxyapatite—nanoHA), and the other 24 received the same mini implants with L-PRF, which was collected by cardiac puncture, centrifugated, and inserted in the bone bed. The animals were euthanized 7 and 30 days after implant placement, and the retrieved samples were prepared for microtomographic and histomorphometric (bone-to-implant contact—BIC; and Bone Area Fraction Occupancy—BAFO) analyses. The adhesion of the nanoHA surface onto the implant surface was investigated by insertion and removal in simulated bone medium (Sawbones). The adhesion evaluation revealed that the loss of nanoHA after this procedure (as measured with SEM) from the implant surface was less than 1%. Overall, the nanoHA surface presented more bone in contact and in proximity to the implant, a higher bone surface/tissue volume fraction, a higher number of bone trabeculae, as well as trabecular separation relative to the DAE surface. Such results were more evident when the nanoHA surface was combined with L-PRF and after 30 days in vivo. The nanoHA surface presented higher BAFO when compared to DAE, with or without association with L-PRF. Therefore, implants with a nanoHA surface potentially benefit from the association to L-PRF.

## 1. Introduction

The surface morphology and roughness of dental implants are important factors that influence cell proliferation and differentiation, the synthesis of extracellular matrix, the production of local growth factors, and the types of cells on the implant surface [1]. The adhesion of osteoblasts to the implant surface is fundamental to start the cell signaling, stimulating cellular proliferation [1]. The surface roughness of the implants can facilitate the retention of osteogenic cells and allows the migration of this type of cell to the implant surface through osteoconduction [2]. Moreover, it promotes better adhesion of collagen and increases surface area, leading to more sites for cellular fixation, increased tissue growth, and mechanical stability [3]. Due to these advantages, the surface of implants evolved from the machined to the moderately rough textured surfaces with chemical modifications [4,5,6]. Studies using histomorphometry and biomechanical analysis have shown that the use of textured surfaces lead to higher osteoconductivity in comparison with machined implants [5,6].

The introduction of nanotopographic modification to the surface of the implants affects the bone response after implantation [7]. A preclinical rat animal model has been shown to increase early bone formation by using nanohydroxyapatite (nanoHA)-coated implants, which was attributed to the potential chemical bioactivity of the hydroxyapatite and/or the nanostructured topography that facilitates molecular-level interaction with the surface [8]. Similarly, in an in vitro study, Martinez et al. [9] evaluated the behavior of osteoblastic cells in surfaces coated with nanoHA in comparison with a conventional surface treatment with dual acid etching (DAE). In the study, discs of commercially pure titanium were submitted to the surface modification/treatment (nanoHA or DAE) and the cell proliferation and viability along with osteogenic biomarkers (type I collagen and osteopontin) were assessed. The results showed greater morphologic spreading of cells on the nanoHA surface in comparison with the conventional DAE over time. The expression of osteopontin was higher after 24 h on the nanoHA surface in comparison with the DAE surface. After 72 h, the cell proliferation and viability were also higher on the nanoAH surface as well as the expression of type I collagen. Altogether, the data indicate that the nanoHA surface improved the early events of osseointegration. Even in more challenging clinical scenarios, a nanoHA surface might hasten bone regeneration. In a preclinical diabetic rat model, Oliveira et al. [10] evaluated the influence of nanoHA-coated implants on bone formation by assessing osteogenic markers, in which an upregulation of genes associated with bone formation was observed when implants with a nanoHA surface were placed in the diabetic rats.

Other types of surface modifications, new products, and protocols have been investigated for the optimization of osseointegration. One example is the use of platelet concentrates [11]. Platelets contain high amounts of growth factors such as PDGFs (platelet-derived growth factors), TGF-β (transforming growth factor—β) and VEGF (vascular endothelial growth factor) that stimulate cell proliferation, angiogenesis, osteoblastic differentiation and proliferation, and also stimulates the remodeling of the extracellular matrix. Furthermore, the platelets have demonstrated antimicrobial properties [12,13].

Leukocyte–platelet-rich fibrin (L-PRF) belongs to the second generation of platelets which was described in 2001 [14]. L-PRF is processed easily by centrifugation of the blood immediately after it is collected. The blood coagulates resulting in a gel rich in fibrin and leucocytes, without biochemical processing. Therefore, there is virtually no risk of a rejection reaction in the organism [15]. In a preclinical dog animal model, Neiva et al. [16] evaluated the synergetic effect of implant surface texture and the presence of L-PRF on bone healing on immediately placed implants. L-PRF was used in the gap between implant and socket walls on one side of the mandible. The authors reported that the combined use of L-PRF and implants with nanotopographic surface modification increased the bone formation around the implants.

In another study, Öncü et al. [12] evaluated the use of L-PRF to improve tissue healing and osseointegration in rabbits. In total, four defects (5 mm in length and 3 mm in diameter) were created in the tibia of each animal, which were covered with L-PRF along with the implant, which was soaked in the L-PRF previous to installation. Histomorphometry analysis revealed that the use of L-PRF potentially increases the rate of new bone formation during the early healing period and promotes faster osseointegration. Similarly, in a systematic review, Castro et al. [17] analyzed the effect of L-PRF on bone regeneration procedures and osseointegration. The study showed that the use of L-PRF might preserve the alveolar shape, improve the stability of the implants, and lead to less marginal bone loss. In another systematic review, Lyris et al. [18] used meta-analysis to evaluate the use of L-PRF on the stability of dental implants. Three time points were evaluated: immediately after implantation, after one week, and after four weeks. The results suggested that the L-PRF benefits the secondary (biological) implant stability of the implants.

Therefore, considering the development of new implant surfaces and the recent studies showing the benefits of using growing factors, the present in vivo study aimed to evaluate the use of nanohydroxyapatite-coated implants in association or not with L-PRF to promote bone repair and osseointegration.

## 2. Materials and Methods

### 2.1. Ethical Committee

This study was approved by The Ethics Committee on Animal Experimentation at the school of Dentistry of Ribeirão Preto, University of São Paulo, under the protocol number 2018.1.164.58.4. The procedures were initialized after the approval of the committee and according to the ethical norms from the Brazilian College of Animal Experiments (named as COBEA).

### 2.2. Animals

Forty-eight adult rats (Rattus norvegicus albinus, Wistar) weighing 200–250 g were included in this study. The rats were obtained from the central animal facility from the school of Dentistry of Ribeirão Preto, University of São Paulo, and were kept in plastic cages with free access to water and a standard diet. During the experimental period, the rats stayed in the animal facility in an environment with a cycle of 12 h of daylight and a temperature of 22–24 °C.

### 2.3. Implants and Surface Treatments

All the mini-implants were produced by S.I.N Implant System, São Paulo, Brazil. The implants were manufactured from titanium (grade 4) and measured 2.7 mm in length and 1.4 mm in diameter. The dual acid etching was performed using a proprietary process developed by S.I.N that includes baths of nitric acid followed by sulfuric acid, in a microcorrosion process. The nanoHA treatment was performed by applying a coating liquid consisting of a dispersion of 1% nanohydroxyapatite crystals, 43% p-xylene (99%, Aldrich), 50% Pluronic L64 surfactants (BASF), and 6% water (Type 1) which was added on top of the implant to be coated, and the implant was placed on a device which enabled the controlled rotation of the implant. The implant was spun at 2600 rpm for 3 s, which created an even layer of coating liquid on the substrate. The implant was then allowed to dry for 10 min at room temperature. The implant was then placed in an oven at 450 °C for 5 min. This heat treatment had the purpose of sintering and improving the adhesion of the HA crystals. The treatment resulted in an evenly dispersed layer of needle-shaped HA crystals on the implant surface, 20–50 nm long and approximately 5 nm in thickness. The crystal layer was around 20–40 nm thick. A more detailed description of the chemical composition, adhesion strength, and biological performance of the coating can be found in Johansson et al., 2015 [19].

### 2.4. Adhesion Testing

The implants used in the adhesion testing were manufactured from titanium (grade 4) by S.I.N. Implant Systems and had a diameter of 3.5 mm and a length of 10 mm. The surface treatment was identical to the mini-implants used in the study, i.e., with a DAE surface followed by nanoHA treatment. In total, five implants were used in the test. Three implants were inserted into a synthetic bone medium consisting of a rigid polyurethane foam (Sawbones 40 PCF, Sawbones Europe AB, Malmö, Sweden). Holes with a diameter of 3.2 mm and a depth of 20 mm, approximately 30 mm apart, were drilled in the Sawbones material. The implants were then inserted in the holes until the upper part of the implant was level with the Sawbones material. The insertion torque during the insertion and removal was measured with a Tohnichi BTG90CN torque meter. After insertion, the implants were unscrewed from the Sawbones block, and any loosely attached Sawbones particles were removed using compressed air. To remove the remaining Sawbones, the implants were then placed in separate vials, each containing 15 mL of ethylenediamine (99+%, Thermo Scientific), and sonicated in an ultrasonic bath (Bandelin Sonorex RK 156 BH) for approximately 80 min. After the ultrasonic treatment, the implants were placed in separate beakers, each containing 100 mL of 2-propanol (ACS grade, Fisher Chemicals), for approximately 4 h, followed by drying in a compressed air stream for approximately 1 min. Two implants served as controls and were only subjected to ultrasonic cleaning in ethylenediamine, washing in 2-propanol, and drying in compressed air.

**Figure 1:**
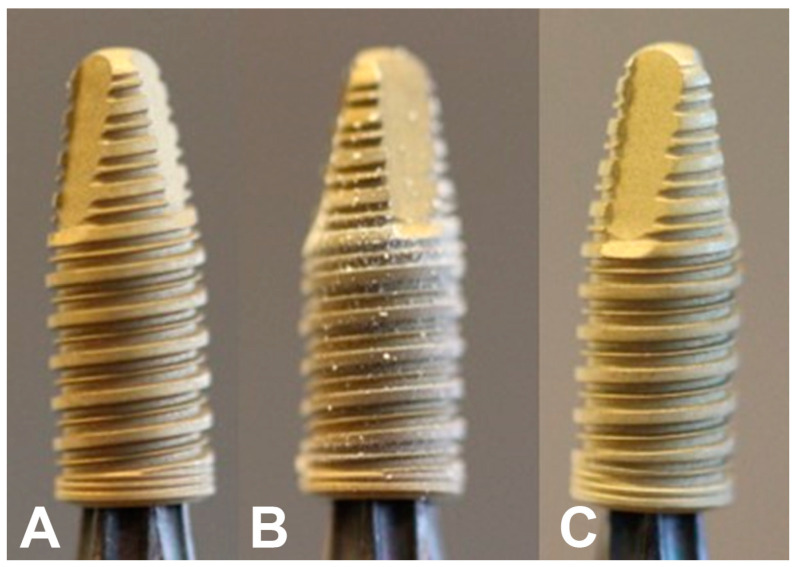
(**A**) Implant before insertion in Sawbones, (**B**) after Sawbones insertion and removal, showing visible Sawbones residue, and (**C**) after insertion, removal, and cleaning.

Figure 1 shows photographs of implants during the different stages of the Sawbones procedure. All implants were analyzed before and after Sawbones testing with a scanning electron microscope (SEM, Zeiss Sigma 300). SEM images were analyzed with the ImageJ software (National Institutes of Health, Bethesda, MD, USA) to estimate the degree of removal of the nanoHA crystal layer.

### 2.5. Preparation of Platelets Concentrates—L-PRF

**Figure 2:**
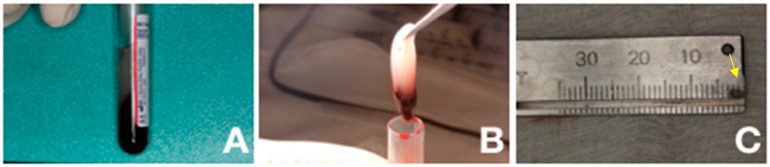
Preparation of platelets concentrates. (**A**) Blood sample in the test tube after centrifugation. (**B**) L-PRF obtained as a membrane. (**C**) Measurement of the membrane—yellow arrow (3 mm).

The preparation of L-PRF in this study was carried out according to the protocol proposed by Choukroun et al. [14], in 2001, standardized by Miron et al. [20], and characterized by da Silva et al., 2022, which observed a higher concentration of both leukocytes and platelets in the buffy coat layer of the L-PRF tube obtained from rat blood samples [21]. Briefly, cardiac puncture was used to collect 500 µL of blood from each animal. The blood was transferred to a sample tube (Figure 2A). In the L-PRF group, immediately after the blood collection, the blood was centrifuged at 2700 rpm for 12 min (relative centrifugal force maximum − RCF-max = 701 g) in the Intra-Spin™ fuge (33° rotor angulation, 55 mm radius at the clot, 86 mm at the maximum, Intra-Lock^®^ International, Inc, Boca Raton, FL, USA). After centrifugation, it was possible to observe three different layers: one more superficial, corresponding to the acellular plasma; a lower layer, corresponding to red blood cells; and an intermediate one, corresponding to the L-PRF matrix (Figure 2B). The matrix measured 3 mm as shown in Figure 2C. The sham operation was conducted in the groups of animals without L-PRF preparation. Animals were anesthetized, and the blood was collected in the same way previously described for L-PRF groups, but the blood was discharged and L-PRF was not prepared.

### 2.6. Surgical Procedures

Initially, the animals were weighed for the correct administration of the anesthetics. General anesthesia was induced by administering an injection of 0.08 mL/100 g of ketamine hydrochloride (Agener, União Ltd.a, São Paulo, Brazil) and 0.04 mL/100 g xylazine hydrochloride (Rompum; Bayer SA, São Paulo, Brazil). After that, the animals were placed in the supine position. Trichotomy and antisepsis were performed using fine-tipped scissors and a 1% PVPI solution, respectively.

**Figure 3:**
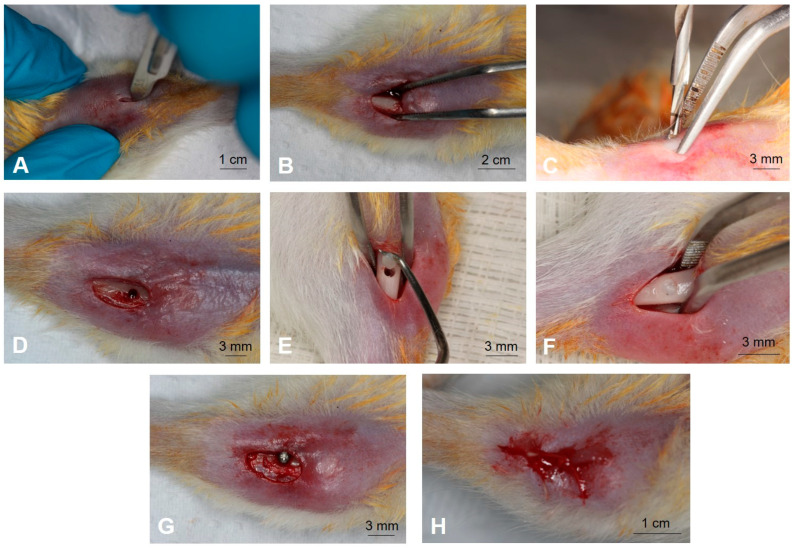
Surgery for implant installation. (**A**) Incision measuring approximately 2 cm, parallel along the axis of the tibia. (**B**) Exposed bone tissue after full-thickness flap. (**C**) Osteotomy being performed. (**D**) Bone before addition of L-PRF. (**E**) Addition of L-PRF. (**F**) L-PRF in the cavity. (**G**) Implant installed. (**H**) Flap sutured.

Subsequently, an incision of 2 cm was made parallel to the axis of the tibia (Figure 3A). The location for the incision was chosen considering the most voluminous portion of the bone tissue selected by palpation. The muscle tissue was sectioned until the periosteum was exposed, using a No.3 scalpel handle, mounted with a No. 15 scalpel blade (Swann-Morton, Sheffield, UK).

The detachment was performed using the Freer and Molt detachers and with an Adson forceps with teeth. These same instruments, together with anatomical forceps, helped to move the flap and stabilize the tibia (Figure 3B). As the procedure in this region does not cause excessive bleeding, the surgical area was dried with sterile gauze.

The osteotomy for implant placement was performed with an appropriate drill under constant irrigation with saline solution, as recommended by the manufacturer (S.I.N.—Implant System, São Paulo-SP) (Figure 3C,D). Prior to implant placement, in the groups associated with L-PRF, the L-PRF was introduced into the prepared bone site (Figure 3E,F). The implants were then installed so that the threads were completely inserted into the cortical bone (Figure 3G). The surgical procedure ended with the primary closure of the tissues using sutures in layers (Figure 2H), using absorbable sutures (Vicryl Ethicon 5.0, Johnson Prod., São José dos Campos, Brazil).

After the surgery, the animals received a single dose of antibiotics (penicillin 24.000 IU/kg; 0.01 mL/100 g of animal weight via intramuscular—Pentabiótico Veterinário Pequeno Porte, Fort Dodge^®^ Saúde Animal Ltda., Campinas, SP, Brazil), anti-inflammatory (Buprenorfin 0.3 mg/mL; 0.05 mg/kg of animal weight—subcutaneous injection, 12/12 h), and also an analgesic (Flunixin, 0.2 mL/kg of animal weight, 12/12 h—subcutaneous injection). The animals were not submitted to any movement or food restriction after the surgery, and they were kept in plastic cages during the experimental period.

### 2.7. Experimental Groups

The 48 animals were randomly distributed in 4 experimental groups, each one containing 12 rats:

- Group 1 (*n* = 12)—nanoHA surface—mini-implants, treated with nanoHA.
- Group 2 (*n* = 12)—nanoHA surface + L-PRF—mini-implants treated with nanoHA, with L-PRF added prior to the insertion of the implant.
- Group 3 (*n* = 12)—dual acid etching (DAE) surface—mini-implants, surface treated with dual acid etching.
- Group 4 (*n* = 12)—dual acid etching (DAE) surface + L-PRF—mini-implants, surface treated with dual acid etching, and with L-PRF added prior to the insertion of the implant.

### 2.8. Euthanasia and Removal of Implants

The animals were euthanized 7 and 30 days after the installation of the implants (24 animals in each time interval, 6 from each experimental group), as previously described in the literature [10,21]. This was performed with an overdose of intraperitoneal anesthetic (sodium thiopental 150 mg/kg of animal weight—Thiopentax^®^, Cristália Produtos Químicos Farmacêuticos Ltda., São Paulo, Brazil). After confirmation of the death of the animals, the right tibias were removed and fixed for analysis of the tridimensional structure using microcomputerized tomography (micro-CT) and histological processing for the histomorphometry analysis.

### 2.9. Micro-CT Analysis

After 48 h of fixation in 10% buffered formalin, the samples were scanned using the high resolution microtomograph Skyscan 1172–160 micro-CT (Bruker, Kontich, Antwerp, Belgium) to obtain tomographic bidimensional projections and 3D reconstruction. For the scanning, aluminum-copper was used to increase contrast, the pixel size used was 5.87 μm, rotation of 360°, and a rotation step of 0.40. The voltage and current were set to 100 kV and 100 μA, respectively.

The tomographic bidimensional projection and 3D reconstruction were performed using NRcon software (NRecon v.1.6.10.4, Bruker, Kontich, Antwerp, Belgium), and the implants were positioned along their longest axis using the DataView software (v.1.5.0, Bruker, Kontich, Antwerp, Belgium). The sagittal axis was selected for the complete visualization of the implants.

Subsequently, the reconstructions were submitted to morphometric analysis using CT analyzer software (CTAn., v.1.15.4.0, Bruker, Kontich, Antwerp, Belgium) for evaluation of the bone around the implant according to the following tomographic parameters: microtomographic bone-to-implant contact = IS/TS, percent; bone volume fraction = BV/TV, percent; trabecular number = Tb.N; trabecular separation = Tb.Sp, mm.

For the CTAn, custom processing was used. The measurements were performed from the coronal area of the implant and reaching its entire length. The Regions of Interest (ROI) were determined, and the Volume of Interest (VOI) was determined by the integration of all the ROIs across all the selected image levels. The VOI represents the 3D volume selected. The transformation into binary code was performed using the gray scale defined by a density of 35–150 for bone and 150–255 for implant. All micro-CT analyses were performed by a single examiner, blinded to the experimental groups.

### 2.10. Sample Preparation for Histomorphometry

After the microtomography, with the implants installed, the tibias were prepared for the histomorphometry evaluation. Each sample was placed in a glass vial containing the 4% formalin in sodium phosphate buffer (PBS) at pH 7 for 10 days at room temperature. Afterwards, the samples were transferred to ethanol solution 70% for 72 h and then dehydrated in increased concentrations of ethanol gradient (solutions 70%, 95% and 100%). After dehydration, the tibias were embedded in resins, Hard Grade LR White (London Resin Company, Berkshire, England). The samples embedded in resin were sectioned using the Exakt Cutting System (Exakt, Norderstedt, Hamburg, Germany) using the hard tissue sectioning technique described by Donath and Breuner, 1982. Then, the Exakt Grinding System (Exakt, Norderstedt, Hamburg, Germany) was used to polish the longitudinal sections of approximately 50 to 80 µm, which were mounted on acrylic slides. The sections were assembled in histological blades for the analysis, being stained with Stevenel’s blue and Alizarin red S.

### 2.11. Histomorphometric Analysis

A longitudinal section of 50–80 µm thickness of each implant was captured with a camera, Leica DC 300F (Leica Microsystems GmbH, Nussloch, Germany), attached to the stereomicroscope Leica MZFL III (Leica Microsystems GmbH, Nussloch, Gemany). The images were analyzed using ImageJ to determine the percentage of Bone-to-Implant Contact and the bone density (percentage of trabecular bone area in relation to the total bone area) in the adjacent and distant areas of the implants.

**Figure 4:**
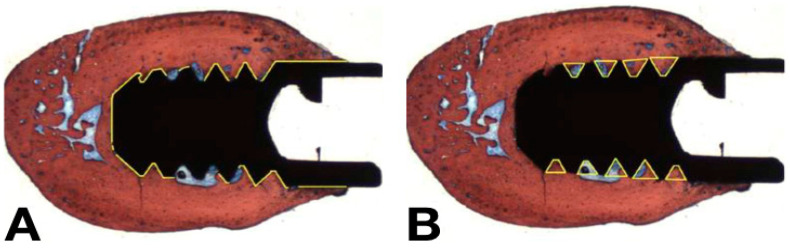
Schematic illustration of the measurements of (**A**) BIC and (**B**) BAFO.

The histomorphometric analyses were also conducted using ImageJ in order to quantify and evaluate the parameters of osseointegration around the peri-implant surface. The bone-to-implant contact (BIC) was used to quantify the degree of osseointegration derived from primary stability by measuring the percentage of the bone in contact with the perimeter of the implant surface (Figure 4A). The Bone Area Fraction Occupancy—BAFO) was used to evaluate the degree of osseointegration derived from secondary stability by measuring the percentage of bone inside the threads of the implants (Figure 4B). All the analyses were performed by a single examiner, blinded to the experimental groups.

### 2.12. Statistical Analysis

All the variables are presented as a function of mean values with the corresponding 95% confidence interval (mean ± 95% CI). Preliminary analyses have shown indistinguishable variances (Levene’s test, all *p* > 0.25). Additionally, the data were collected and aligned along a linear mixed model with fixed factors of time (7 and 30 days), surface modifications (nanoHA surface and DAE), and L-PRF (present or not). The Tukey’s test was used for multiple comparisons. The analysis was accomplished using SPSS (IBM SPSS 23, IBM Corp., Armonk, NY, USA).

## 3. Results

### 3.1. Adhesion Testing

**Figure 5:**
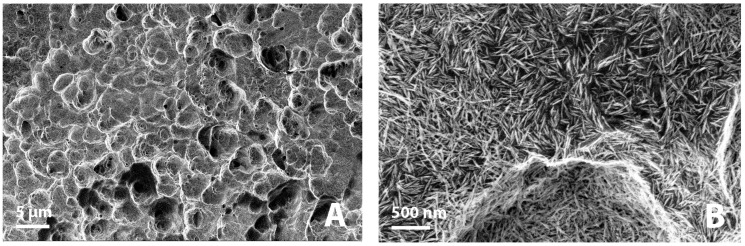
(**A**) SEM image of the nanoHA surface before Sawbones insertion (scale bar = 5 µm), (**B**) high magnification SEM image of the nanoHA surface before insertion (scale bar = 500 nm).

**Figure 6:**
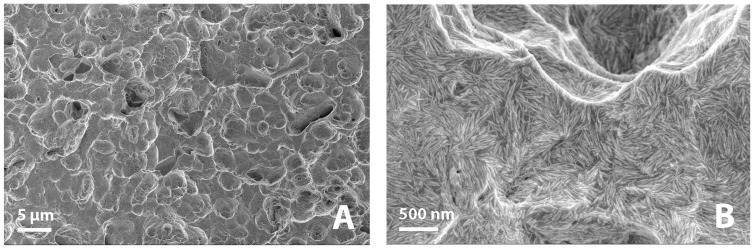
(**A**) SEM image of the nanoHA surface after Sawbones insertion (scale bar = 5 µm), (**B**) high magnification SEM image of the nanoHA surface after Sawbones insertion (Scale bar = 500 nm).

The torque increased linearly during the insertion of each implant and peaked at 35 Ncm. Figure 5A,B shows the representative low and high magnification SEM images of the implant surfaces before insertion in Sawbones. As seen from Figure 5A, the HA coating was evenly distributed on the implant surface and followed the underlying microstructure. At higher magnification (Figure 5B), the needle-shaped HA crystals are clearly visible. Figure 6A,B shows the representative low and high magnification SEM images of the implant surfaces after the insertion, removal, and cleaning procedure. Residues from the polymeric Sawbones material were observed on a few areas on the implants, but the amount was very low overall. SEM examination of the implants which had been cleaned with ethylenediamine and 2-propanol but which had not been subjected to the Sawbones procedure showed no damage to the HA crystal layer. The cleaning procedure was, therefore, successful in removing the Sawbones residue without damaging the HA coating. As seen from Figure 5 and Figure 6, there is very little difference between the nanoHA surface before and after the Sawbones procedure. Removal of HA crystals was observed on a few areas on the thread tops, but the total extent of the coating loss was very low. An image analysis on randomly selected SEM images showed that the degree of removal of HA crystals (i.e., areas without HA/total surface area) was less than 1%.

### 3.2. Surgery

There were no complications regarding the surgical procedure with the animals. The rats recovered consciousness around 30 min after the surgical procedure, and there was no complication in the postoperation period. Additionally, no adverse events such as implant exposure or cardiac alterations were observed. A clinically healthy soft tissue appearance was observed throughout the study.

### 3.3. Micro-CT

**Figure 7:**
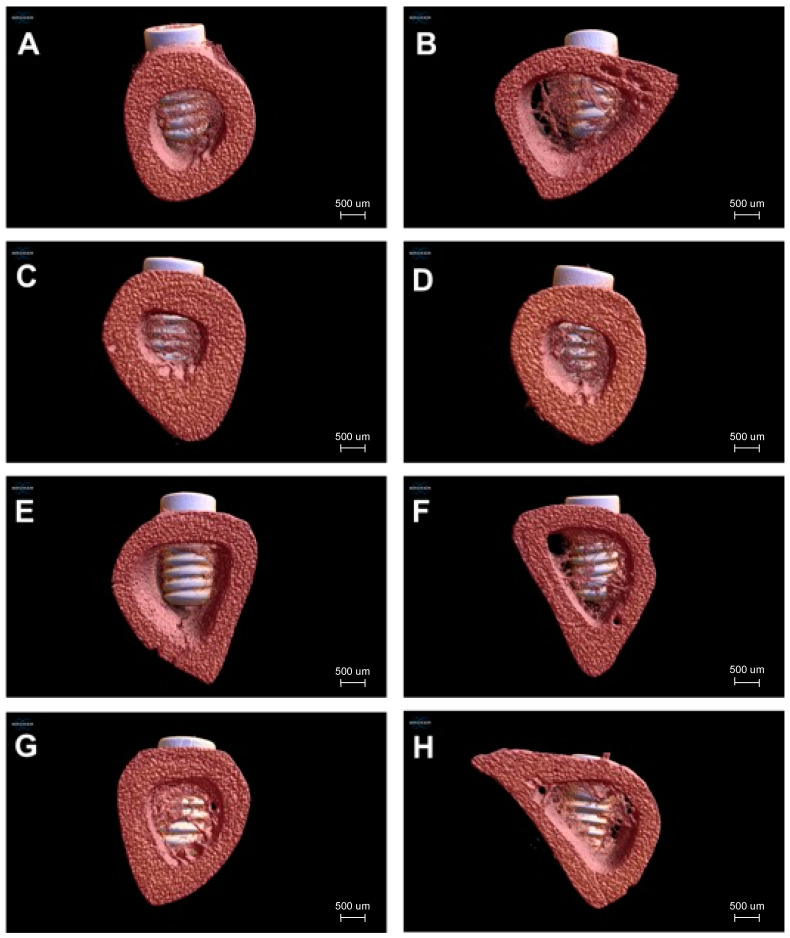
Microtomographic 3D reconstructed images from (**A**) Group 1 after 7 days, (**B**) Group 1 after 30 days, (**C**) Group 2 after 7 days, (**D**) Group 2 after 30 days, (**E**) Group 3 after 7 days, (**F**) Group 3 after 30 days, (**G**) Group 4 after 7 days, (**H**) Group 4 after 30 days.

The microtomographic 3D reconstructed images from all the experimental groups are shown in Figure 7. In analyzing the images, it is possible to observe a successful bone repair irrespective of the time, type of implant surface, and the use or not of L-PRF. Overall, an increase in bone formation was seen from 7 to 30 days. It was also observed that Group 2 (NanoHA surface + L-PRF) showed higher bone formation when compared to the DAE-surface in association or not with the presence of L-PRF. It should be noted that the nanoHA coating itself is not visible with micro-CT, the coating is too thin to be analyzed with this technique.

### 3.4. Microtomographic Bone-to-Implant Contact (IS/TS)

**Figure 8:**
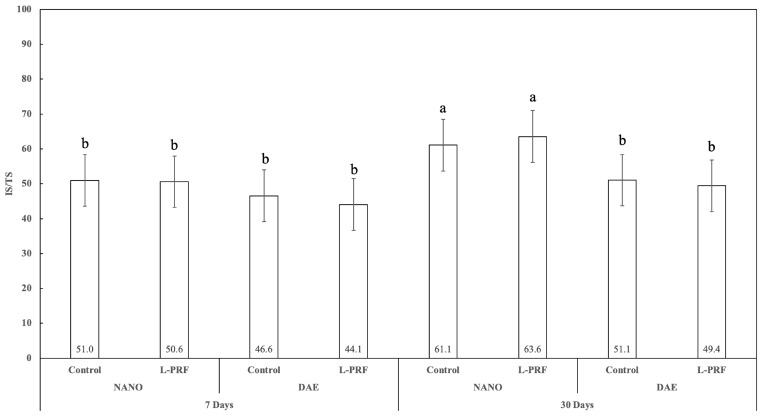
Graphic showing IS/TS percent as a function of all the study factors (time, implant surface, and presence or not of L-PRF) (average ± 95% CI). Different letters indicate statistically significant difference (*p* < 0.05).

The percentage of microtomographic bone-to- implant contact (IS/TS, intersection bone to implant surface/total implant surface) showed a trend to present higher values after 30 days of healing relative to 7 days, with statistically significant differences between 7 and 30 days for implants with the nanoHA surface, regardless of L-PRF use (*p* < 0.016). At 7 days, no statistically significant difference was observed between nanoHA and DAE surfaces either with or without the presence of L-PRF (*p* > 0.214). In contrast, at 30 days, a statistically significant difference was observed in the IS/TS values between the nanoHA and DAE surfaces (*p* < 0.009). In addition, the use of L-PRF resulted in the absence of statistically significant differences for all pairwise comparisons of IS/TS values for timepoints and implant surface types (*p* > 0.631) (Figure 8).

### 3.5. Bone Volume Fraction (BV/TV)

**Figure 9:**
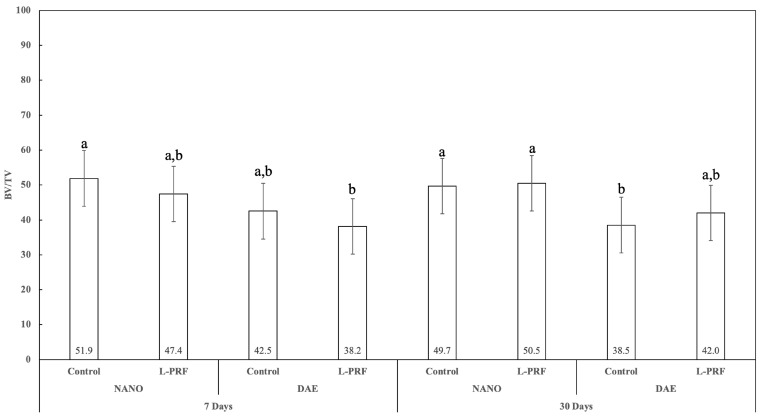
Graphic showing BV/TV percentas a function of all the study factors (time, implant surface, and presence or not of L-PRF) (average ± 95% CI). Different letters indicate statistically significant difference (*p* < 0.05).

The percentage of bone volume fraction (BV/TV) as a function of time, implant surface, and presence or not of L-PRF presented a trend of showing better results with the use of the nanoHA surface in comparison with DAE surface at 7 and 30 days of the study for both groups, using or not using L-PRF; with statistically significant differences between the nanoHA and DAE surfaces without the presence of L-PRF at 30 days (*p* < 0.050). No statistically significant difference was observed between 7 and 30 days as well as with the presence or not of L-PRF pairwise comparisons (*p* > 0.429) (Figure 9).

### 3.6. Trabecular Separation (Tb.Sp)

**Figure 10:**
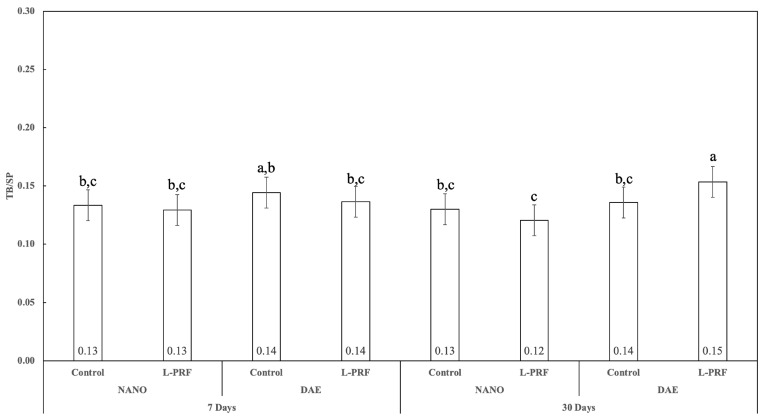
Graphics showing Tb.Sp as a function of all the study factors (time, implant surface, and presence or not of L-PRF) (average ± 95% CI). Different letters indicate statistically significant difference (*p* < 0.05).

Trabecular separation (Tb.Sp) showed no statistically significant difference for all 7 and 30 days of healing pairwise comparisons (*p* > 0.336), except for DAE surface implants with the presence of L-PFR (*p* < 0.050). At 7 days, no statistically significant difference was observed between the nanoHA and DAE surfaces either with or without L-PRF (*p* > 0.247). In contrast, at 30 days, a statistically significant difference was observed in the Tb.Sp results between the nanoHA and DAE surfaces when L-PRF was used (*p* < 0.001). The use of L-PRF resulted in no statistically significant difference in the Tb.Sp values after 7 days of healing, irrespective of implant surface type (*p* > 0.406); however, at 30 days, the use of L-PRF significantly increased Tb.Sp values for implants with the DAE surface (*p* < 0.050) (Figure 10).

### 3.7. Trabecular Number (Tb.N)

**Figure 11:**
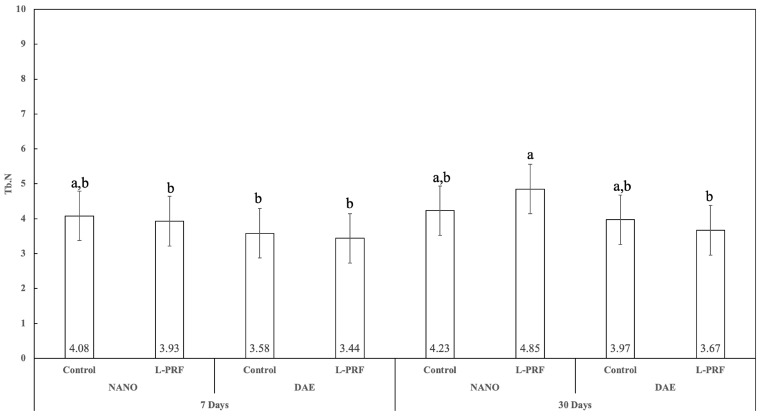
Graphics showing Tb.N as a function of all the study factors (time, implant surface, and presence or not of L-PRF) (average ± 95% CI). Different letters indicate statistically significant difference (*p* < 0.05).

The trabecular number (Tb.N) showed no statistically significant difference for all 7 and 30 days of healing pairwise comparisons (*p* > 0.437), except for the comparison of DAE surface implants with the presence of L-PFR (*p* < 0.050). At 7 days, no statistically significant difference was observed between the nanoHA and DAE surfaces either with or without L-PRF use (*p* > 0.321). In contrast, at 30 days, a statistically significant difference was observed in the Tb.N results between the nanoHA and DAE surfaces when L-PRF was used (*p* < 0.022). The use of L-PRF resulted in no statistically significant difference for all Tb.Sp pairwise comparisons for both timepoints and implant surface types (*p* > 0.217) (Figure 11).

### 3.8. Histomorphometric Results

**Figure 12:**
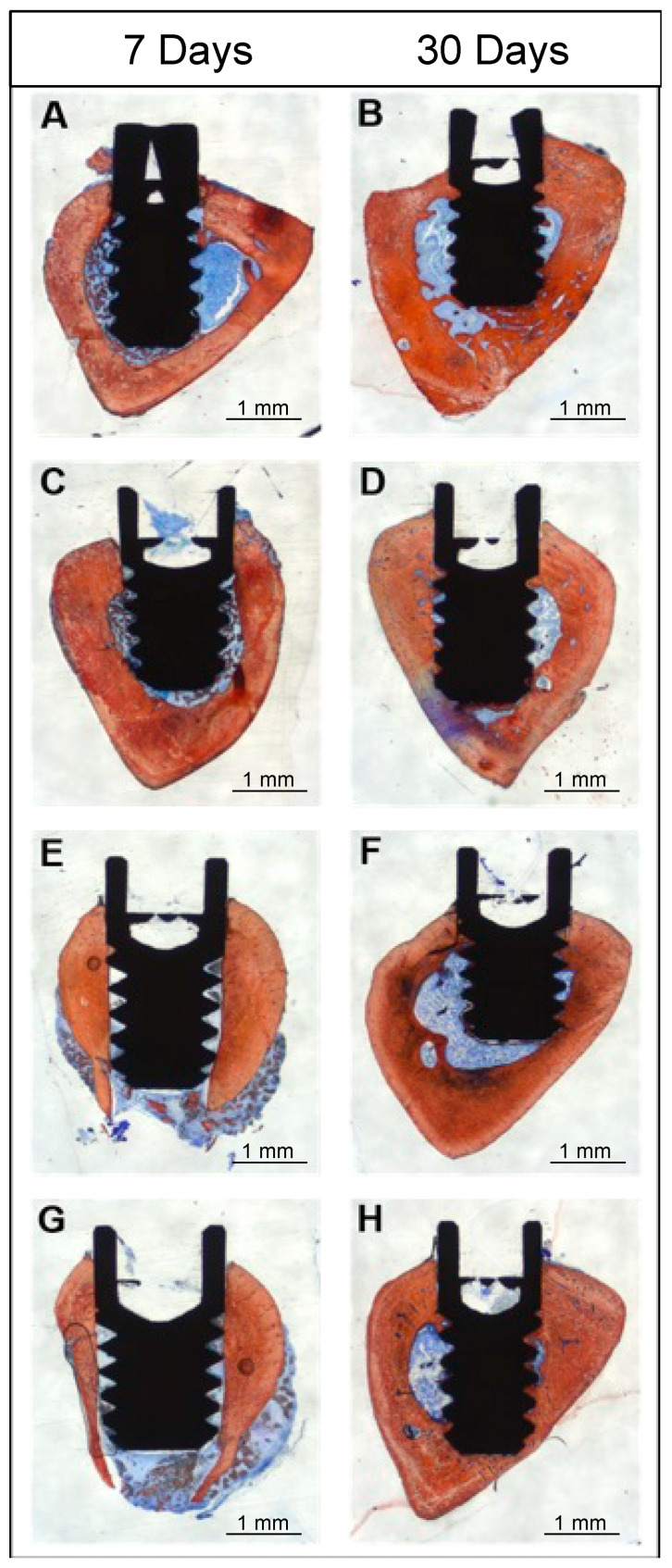
Histologic micrographs of all experimental groups at 7 days (**A**,**C**,**E**,**G**) and 30 days (**B**,**D**,**F**,**H**). Stevenel’s-blue- and Alizarin-red-stained histologic micrographs of NanoHA Group 1 (**A**,**B**); NanoHA + L-PRF group 2 (**C**,**D**); DAE Group 3 (**E**,**F**); DAE + L-PRF Group 4 (**G**,**H**).

A qualitative evaluation of the histologic micrographs of all groups is shown in Figure 12A–H. All images confirmed successful osseointegration, irrespective of time, implant surface, and presence or not of L-RPF. Overall, there was significant difference observed between 7 and 30 days for BIC and BAFO. When comparing the implant surface, the nanoHA group showed higher bone density in comparison with the DAE surface. As seen, the nanoHA coating is not visible in these images. This is due to its thickness (20–40 nm), which is beyond the diffraction limit of an optical microscope.

### 3.9. Bone-to-Implant Contact (BIC)

**Figure 13:**
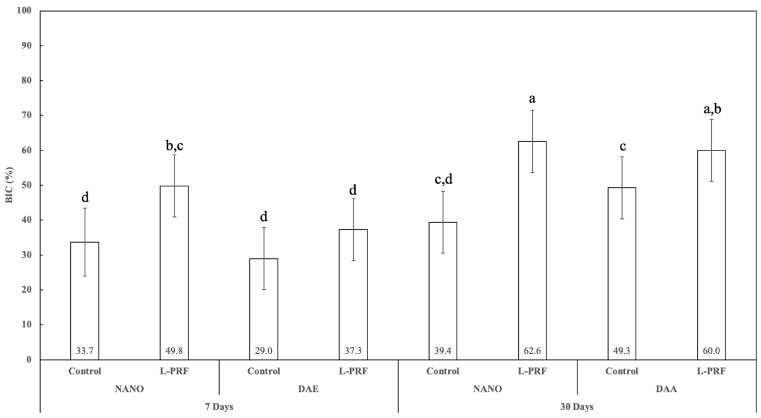
Graphic showing BIC percent as a function of all the study factors (time, implant surface, and presence or not of L-PRF) (average ± 95% CI). Different letters indicate statistically significant difference (*p* < 0.05).

Histomorphometric measurements of the percentage of bone-to-implant contact (BIC) showed statistically significant differences in the percentage for all 7 and 30 days pairwise comparisons (*p* < 0.047), except for the nanoHA surface without L-PRF use (*p* = 0.388). At 7 days, there was no statistically significant difference between the nanoHA and DAE surfaces when L-PRF was not used (*p* = 0.472), while the nanoHA surface presented significantly higher BIC percentage relative to the DAE surface when L-PRF was used (*p* < 0.050). At 30 days, no statistically significant difference between the nanoHA and DAE surfaces was observed either with or without the presence of L-PRF (*p* > 0.120). L-PRF presence significantly influenced BIC percentage for both the nanoHA and DAE surface pairwise comparisons for both timepoints (*p* < 0.018), except for the DAE surface at 7 days (*p* = 0.188) (Figure 13).

### 3.10. Bone Area Fraction Occupancy (BAFO)

**Figure 14:**
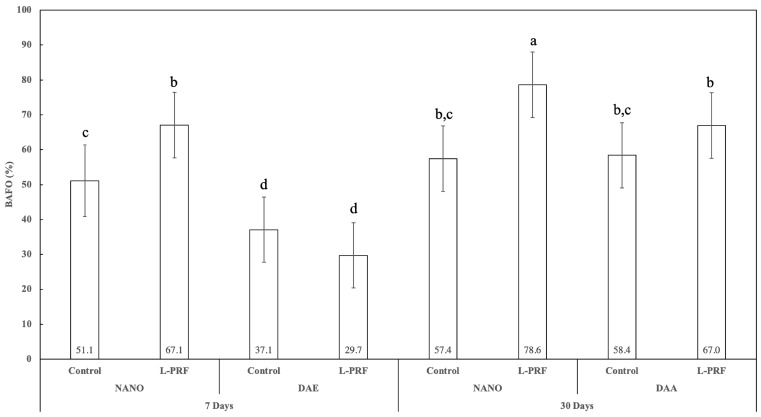
Graphics showing BAFO percent as a function of all the study factors (time, implant surface, and presence or not of L-PRF) (average ± 95% CI). Different letters indicate statistically significant difference (*p* < 0.05).

Histomorphometric measurements of the percentage of bone area fraction occupancy (BAFO) showed statistically significant difference in the percentage for all 7 and 30 days pairwise comparisons (*p* < 0.002), except for the nanoHA surface without L-PRF use (*p* = 0.363). At 7 days, there was statistically significant difference between the nanoHA and DAE surfaces, irrespective of L-PRF use (*p* < 0.048). At 30 days, while no statistically significant difference between the nanoHA and DAE surfaces was observed without the presence of L-PRF (*p* = 0.881), the nanoHA surface outperformed the DAE surface with the presence of L-PRF (*p* < 0.050). While L-PRF presence significantly increased BIC percentage for nanoHA at both 7 and 30 days of healing (*p* < 0.025), L-PRF presence showed no significant influence for the BAFO percentage (*p* > 0.200) (Figure 14).

## 4. Discussion

Implant surface modifications and the use of leukocyte–platelet-rich fibrin (L-PRF) are known strategies to promote osseointegration. In the present study, the in vivo effect of nanohydroxyapatite-coated implants (nanoHA), without or in combination with L-PRF, was investigated. Microtomography and histomorphometric analysis were used for the evaluation of the different responses concerning osseointegration. The results showed that the use of nanoHA-coated implants (nanoHA) led to a higher percentage of bone-to-implant contact (IS/TS) in relation to the dual acid etched surface (DAE), which was statistically significantly different after 30 days of healing. These findings are in accordance with another study performed by de Oliveira et al. [22] that used similar methodology to analyze the benefits of using nanohydroxyapatite-coated implants in healthy and diabetic animals. Jiang et al. [23] also demonstrated that nanoscale surface modifications favor the proliferation and differentiation of osteoblasts.

The percentage of IS/TS was not affected by the association of L-PRF with both implants’ surfaces at 7 and 30 days, in comparison to the correspondent control groups. However, L-PRF use increased BIC and BAFO percentage for both implant surfaces, especially at 30 days of healing. As reported by Castro et al. [17] in a systematic review, the use of L-PRF in the implantation surgery is beneficial for osseointegration. Therefore, the use of L-PRF can be advantageous in association with an implant surface treatment to synergistically maximize the biological potential of the platelet concentrate.

Considering implant surface treatment, the nanoHA showed statistically significant higher values of IS/TS, BV/TV, and BAFO percentages than the DAE surface at the late-healing timepoint. Once more, the result shown in this study corroborates with other studies where nanostructures applied to the implants resulted in a higher degree of osseointegration [7,8,22,23,24]. Mostly, the nanoHA surface IS/TS, BV/TV, and BAFO percentage results were statistically superior to the DAE surface, without or when combined with L-PRF. These results agree with the findings from Ajami et al. [25] who studied osseointegration in a diabetic rat model and the results from Liu et al. [26] and Faverani et al. [27] in studies using an osteoporotic rat model. In such studies, it was concluded that the compromised osseointegration of the implants due to the deficient bone repair of the animals was minimized by the nanotopographic modifications on the implants’ surface with microtopographic complex surface. Furthermore, Strauss et al. [28] demonstrated that the L-PRF can have benefits in osseointegration, ratifying the observations of the present study.

The evaluations of Tb.Sp and Tb.N also revealed statistically better results associated with the nanoHA surface relative to the DAE surface when in combination with L-PRF, highlighting the benefits of this association to the process of osseointegration. In 2020, Canellas et al. [29] performed a clinical study in humans that showed strong evidence of the benefits of using L-PRF in bone formation and to preserve the alveolar ridge. Whilst other studies in the literature reinforced that the chemical surface modification and complex topographic structures, such as implants coated with nanohydroxyapatite, mimic the natural organization of bone tissue and facilitate the interaction with biomolecules and regulate cell behavior during the healing process [30,31,32]. The synergetic effect seen in the present study was also reported by Neiva et al. [16], in a study about dogs, using implants with a nanotexturing together with L-PRF. The authors reported that this combination improved early bone formation.

In the histomorphometric analysis, higher values of BIC and BAFO percentages were found for the nanoHA surface in comparison to the DAE surface, especially when combined with L-PRF. The literature findings indicate that the topography of the implants is fundamental in the early stages of bone regeneration, since the peri-implant bone formation is dependent on the bone’s healing ability [3]. Other strategies to promote faster osseointegration focus on modulating the healing response after the implantation, such as the L-PRF that acts by attracting undifferentiated mesenchymal cells to the site of injury, favoring angiogenesis, chemotaxis, and cell proliferation and, consequently, accelerating the healing and osseointegration process [33]. Öncü et al. [12], in a study using a rabbit model, evaluated if the L-PRF favored the osseointegration and the bone-to-implant contact. The authors observed an increased BIC percentage in the group that used L-PRF and concluded that the presence of L-PRF positively affects healing and bone regeneration in agreement with the findings of the present study using the nanoHA surface.

In the current study, considering the dimensions of the bone preparation in the model used, the osseointegration was already expected in all the groups at 30 days. Nevertheless, the best results were obtained with the association of the nanoHA surface and L-PRF, highlighting the potential benefits of using L-PRF. The observations in this study are similar to other studies in the literature [12,16,27] that report that the use of L-PRF favors the process of osseointegration in many ways and, consequently, decreases rehabilitation time and can influence implant survival.

The benefits of using L-PRF were also demonstrated by Cho et al. [34] who used removal torque to study bone integration in rabbits with artificial bone defects in the tibia. The authors reported that the presence of L-PRF promotes faster osseointegration during the initial healing period. In a systematic review, Castro et al. [17] showed that L-PRF positively affected bone regeneration in surgeries to raise the maxillary sinus. It also influenced the maintenance of the alveolar ridge and bone integration on dental implants. Lyris et al. [18], in another systematic review and meta-analysis, evaluated the influence of L-PRF on the stability of dental implants and concluded that L-PRF improves the secondary (biological) stability and its use can help to accelerate bone healing. These findings corroborate with the results of the present study that showed that BIC and BAFO percentages were statistically higher at 7 days for the nanoHA surface associated with L-PRF, more effectively improving osseointegration already at the early stages of healing.

The experimental model used in the present study indicated that L-PRF was not crucial for osseointegration. However, this model provided a standard bone preparation that allowed us to evaluate and quantify the differences between the experimental groups (the effect of the implant surface treatment and the presence of L-PRF) and its impact on bone-to-implant contact and bone density in the newly formed bone inside the threads. Considering the benefits of the combination of the nanoHA surface and the use of L-PRF, we suggest further clinical studies in animals and humans involving the installation of implants associated with bone defects and/or challenging clinical situations, such as immediate implants, postimplant fenestration, and ridge reconstructions, as well as mechanical tests, such as removal torque, to better evaluate the nature and strongness of the bone regeneration.

## 5. Conclusions

Overall, microtomographic and histomorphometric evaluations showed that implants coated with nanohydroxyapatite (nanoHA) presented more IS/TS, Tb.N, BIC, and BAFO and less Tb.Sp when compared to implants with dual acid etched (DAE) surface treatment, especially after 30 days of healing. It was also revealed that a nanoHA surface benefited more from the association with L-PRF, showing higher values of BIC and BAFO than the DAE surface + L-PRF.

## Data Availability

The data presented in this study are available on request from the corresponding author. The data are not publicly available due to ethical reasons.
